# Supplementary material for: Sulfur stabilizing metal nanoclusters on carbon at high temperatures
Source: Nat Commun. 2021 May 25;12:3135. doi: 10.1038/s41467-021-23426-z (PMC8149400; doi:10.1038/s41467-021-23426-z)
Supplement: Supplementary file 2 — Supplementary Information [file 41467_2021_23426_MOESM2_ESM.pdf]

Supplementary Information for

Sulfur stabilizing metal nanoclusters on carbons at high temperatures

Peng Yin[1]†, Xiao Luo[1, 2]†, Yanfu Ma[1]†, Sheng-Qi Chu[3], Si Chen[1], Xusheng Zheng[4], Junling Lu[1]*, Xiao-Jun Wu[1,2]* and Hai-Wei Liang[1]*

[1]Hefei National Laboratory for Physical Sciences at the Microscale, School of Chemistry and Materials Sciences, University of Science and Technology of China, Hefei, 230026, China.

[2]Synergetic Innovation of Quantum Information & Quantum Technology, CAS Key Laboratory of Materials for Energy Conversion, and CAS Center for Excellence in Nanoscience, University of Science and Technology of China, Hefei, Anhui 230026, China.

[3]Beijing Synchrotron Radiation Facility, Institute of High Energy Physics, Chinese Academy of Sciences, Beijing, 100049, China.

[4]National Synchrotron Radiation Laboratory, University of Science and Technology of China, Hefei, Anhui, 230029, P. R. China

†These authors contributed equally to this work.

*e-mail: hwliang@ustc.edu.cn; xjwu@ustc.edu.cn; junling@ustc.edu.cn

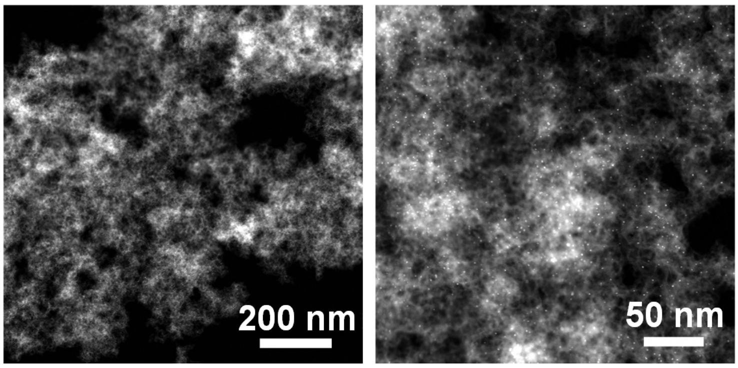

Supplementary Figure 1. Low magnification HAADF-STEM images of the as-prepared Pt/S-C catalyst.

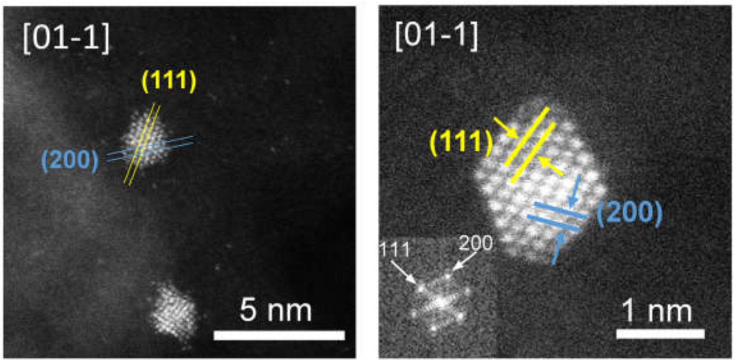

Supplementary Figure 2. Atomic-resolution HAADF-STEM images of Pt/S-C. Pt nanoclusters were well crystalline and mainly enclosed by (111) and (200) crystal planes of face-centered cubic Pt.

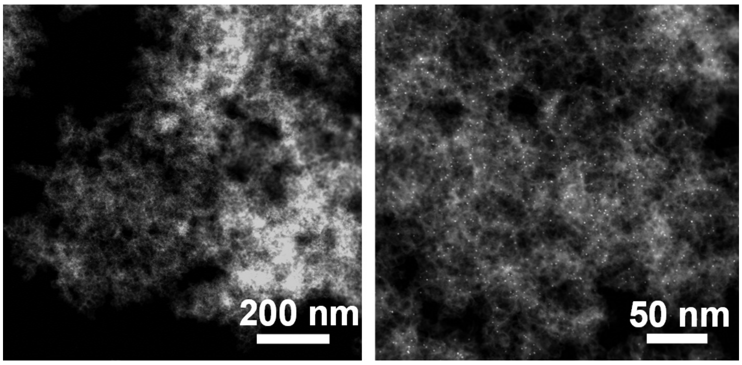

Supplementary Figure 3. Low magnification HAADF-STEM images of Pt/S-C after annealing at 700 °C in 5% H_2/Ar for 600 min.

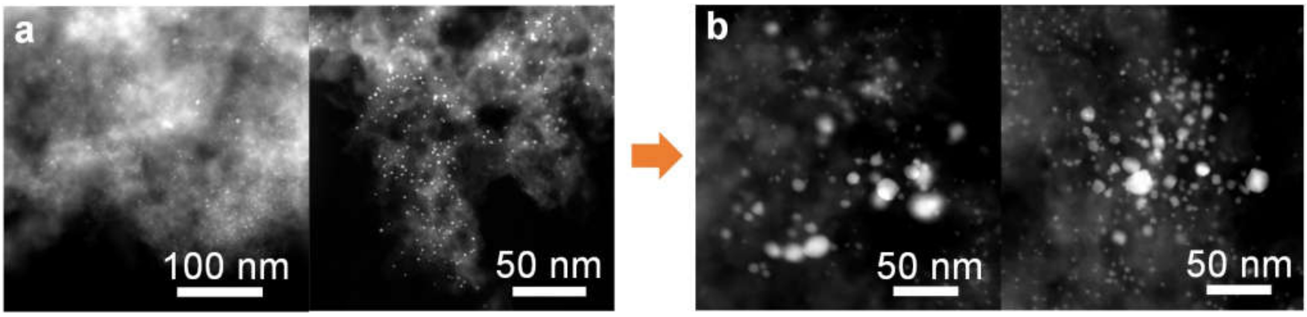

Supplementary Figure 4. HAADF-STEM images of Pt/N-C before (a) and after (b) annealing at 700 °C in 5% H_2/Ar for 600 min. The mesoporous N-C supports were prepared by the cobalt-assisted carbonization of *o*-diaminobenzene, according to our previous works[1,2].

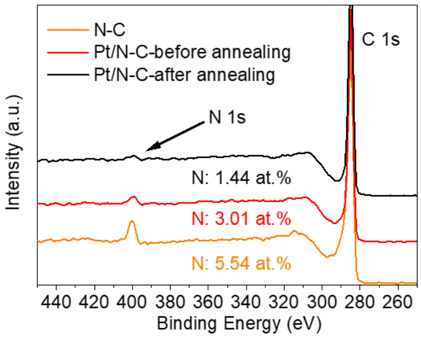

Supplementary Figure 5. XPS survey spectra of the Pt/N-C catalyst before and after annealing treatment at 700 °C in 5% H_2/Ar for 600 min, indicating the significantly decreased nitrogen content from 3.01 to 1.44 at.% after the sintering test.

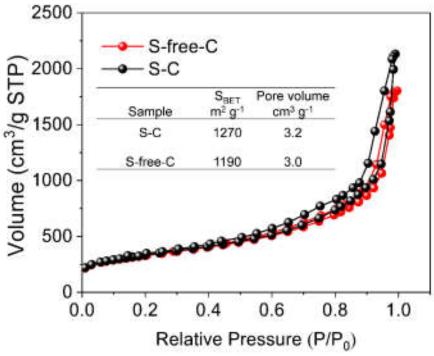

Supplementary Figure 6. N_2 sorption isotherms of S-C and S-free-C, indicating almost unchanged BET surface area and pore volume upon the desulfurization treatment at 1100 °C in 5% H_2/Ar for 120 min.

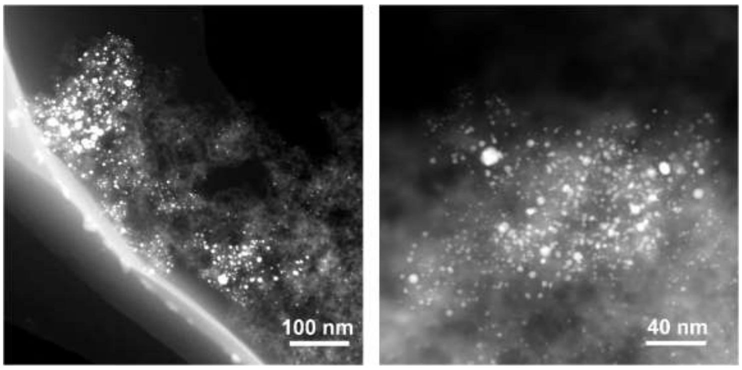

Supplementary Figure 7. Low-magnification HAADF-STEM images of Pt/S-free-C after annealing at 700 °C in 5% H_2/Ar for 600 min.

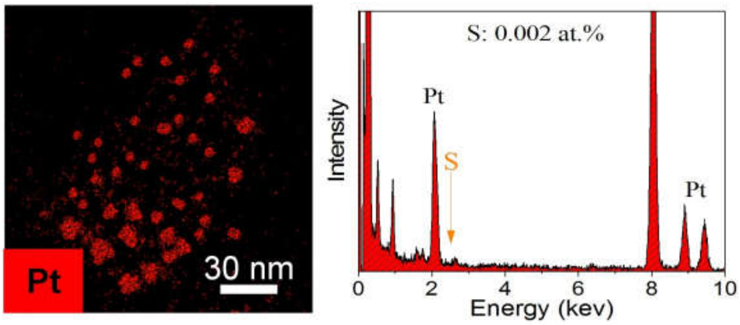

Supplementary Figure 8. EDS elemental mapping of Pt/S-free-C after annealing at 700 °C in 5% H_2/Ar for 600 min, indicating the Pt nanocluster sintering and very low sulfur content.

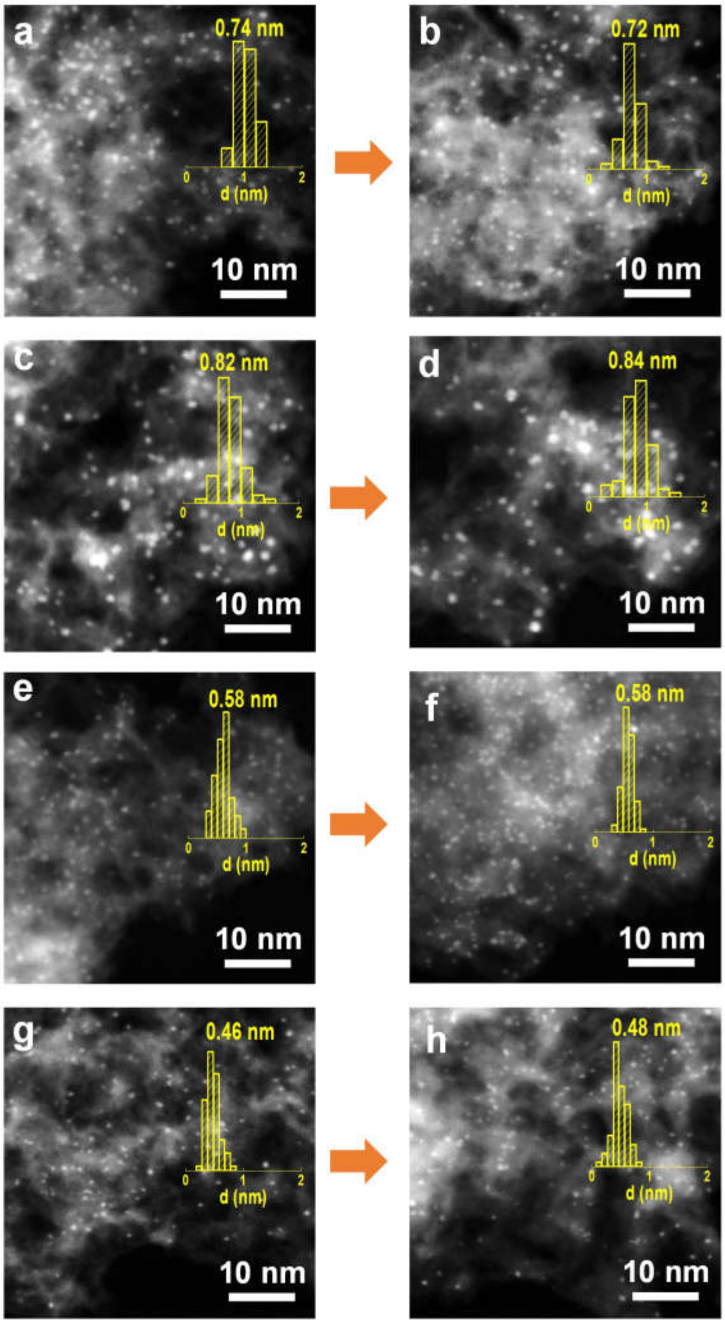

Supplementary Figure 9. HAADF-STEM images and corresponding particles size distribution of Ru/S-C (a, b), Rh/S-C (c, d), Os/S-C (e, f), and Ir/S-C (g, h) before (a, c, e, g) and after (b, d, f, h) annealing at 700 °C in 5% H_2/Ar for 600 min.

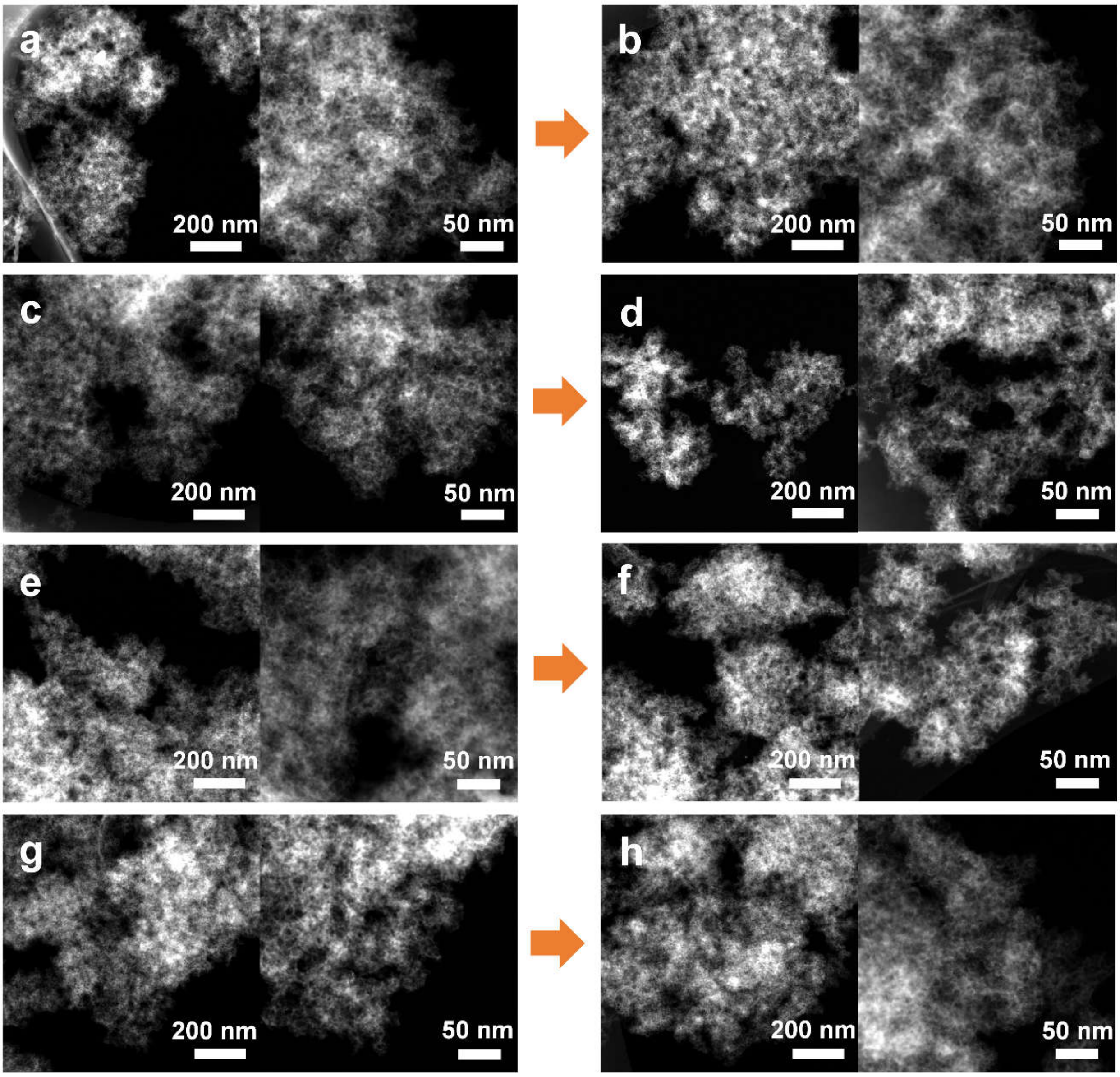

Supplementary Figure 10. Low-magnification HAADF-STEM images of Ru/S-C (a, b), Rh/S-C (c, d) Os/S-C (e, f), and Ir/S-C (g, h) before (a, c, e, g) and after (b, d, f, h) annealing at 700 °C in 5% H_2/Ar for 600 min.

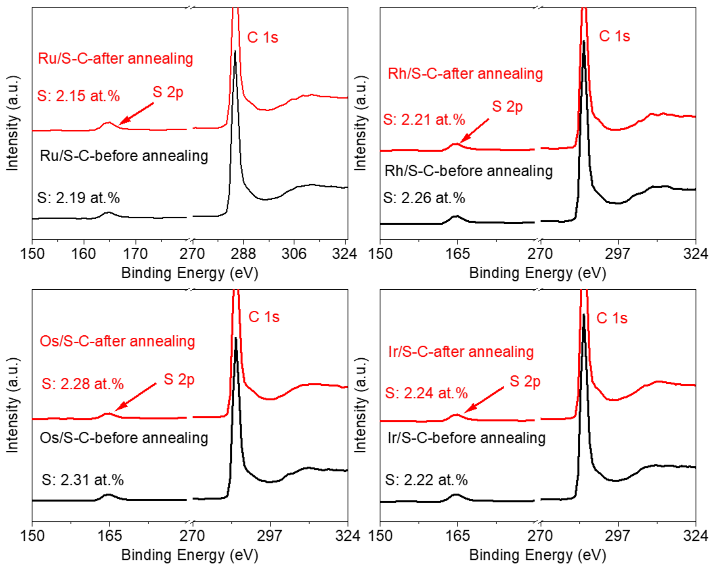

Supplementary Figure 11. XPS survey spectra of Ru/S-C, Rh/S-C, Os/S-C, and Ir/S-C before and after annealing treatments at 700 °C in 5% H_2/Ar for 600 min.

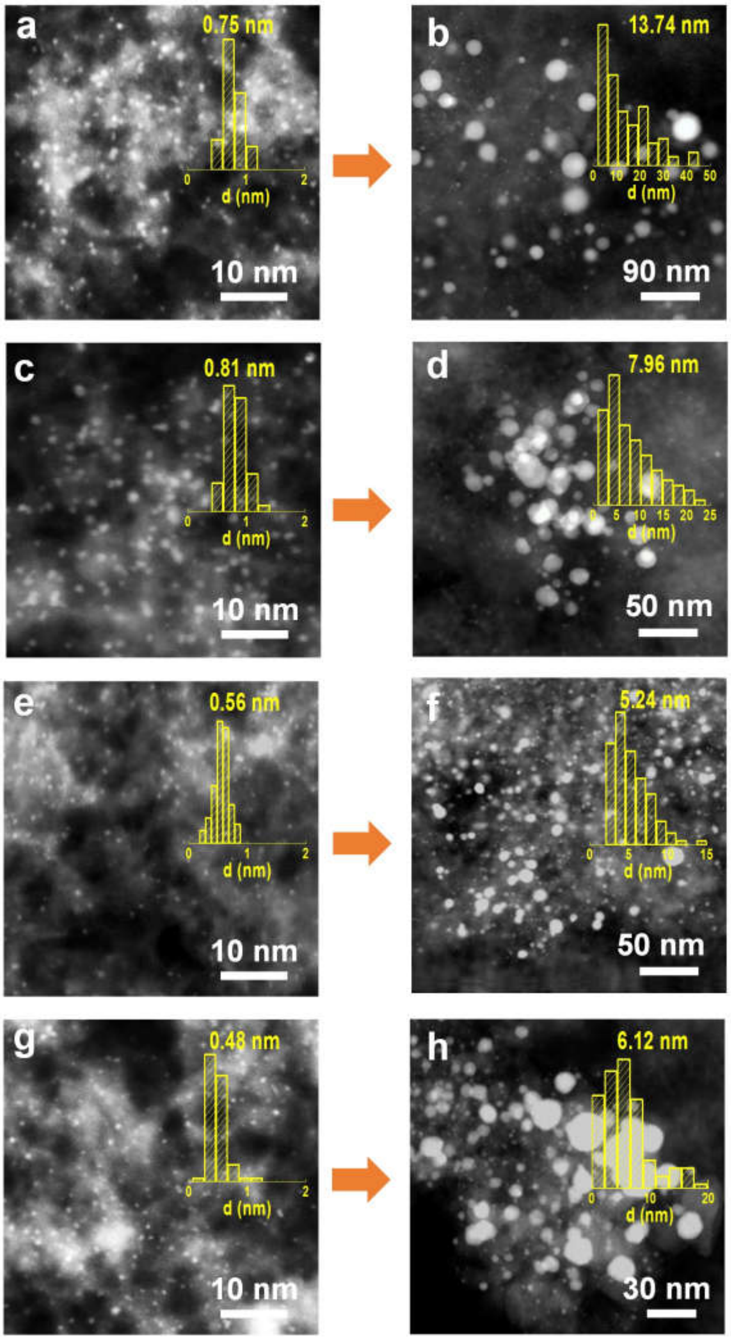

Supplementary Figure 12. HAADF-STEM images of Ru/S-free-C (a, b), Rh/S-free-C (c, d), Os/S-free-C (e, f), and Ir/S-free-C (g, h) before (a, c, e, g) and after (b, d, f, h) annealing treatments at 700 °C in 5% H_2/Ar for 600 min.

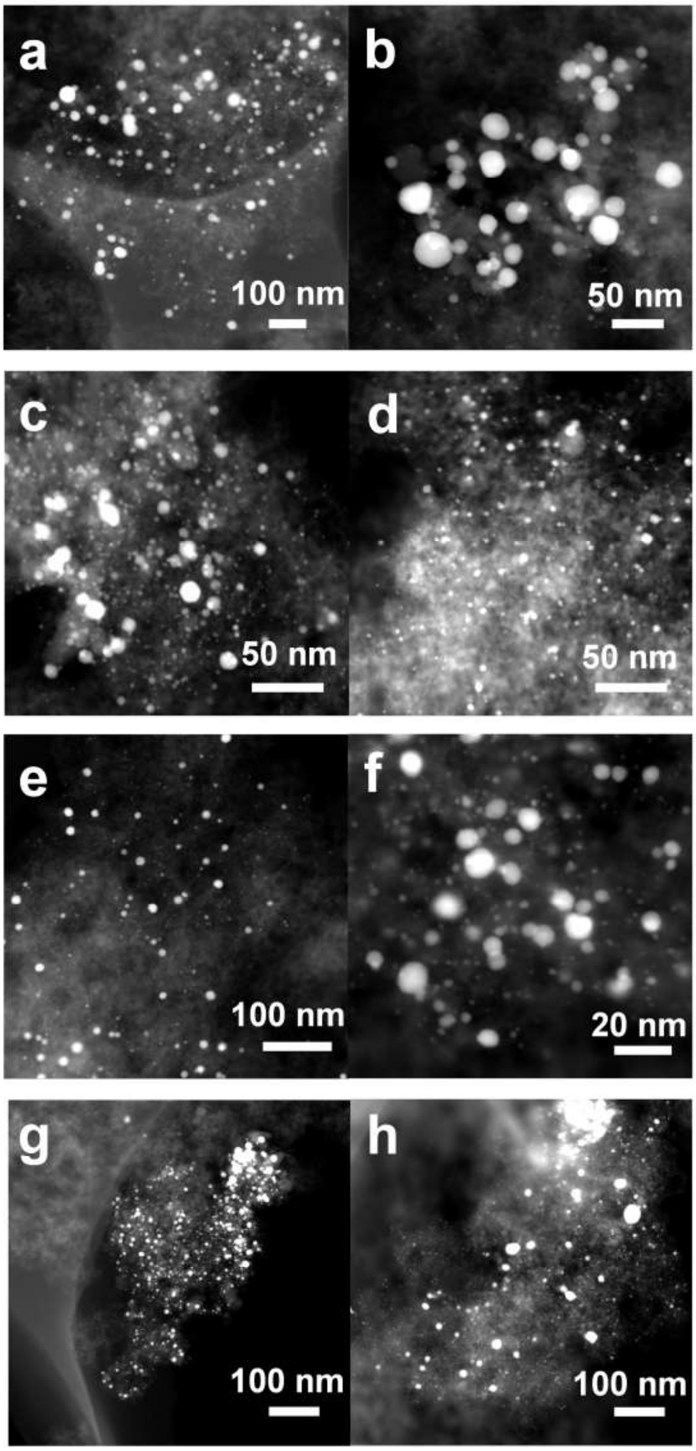

Supplementary Figure 13. Low magnification HAADF-STEM images of Ru/S-free-C (a, b), Rh/S-free-C (c, d), Os/S-free-C (e, f), and Ir/S-free-C (g, h) after annealing treatment at 700 °C in 5% H_2/Ar for 600 min.

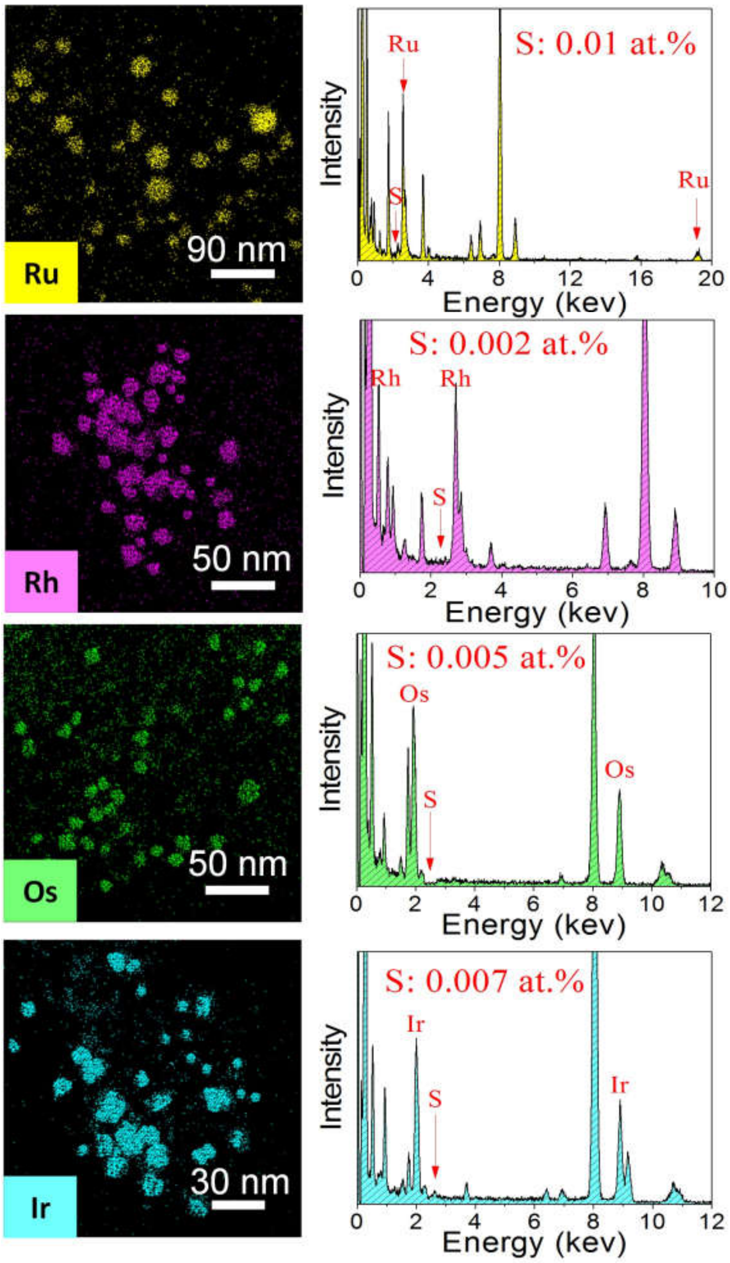

Supplementary Figure 14. EDS elemental mappings of Ru/S-free-C, Rh/S-free-C, Os/S-free-C, and Ir/S-free-C after annealing at 700 °C in 5% H_2/Ar for 600 min, indicating the metal sintering and very low sulfur content.

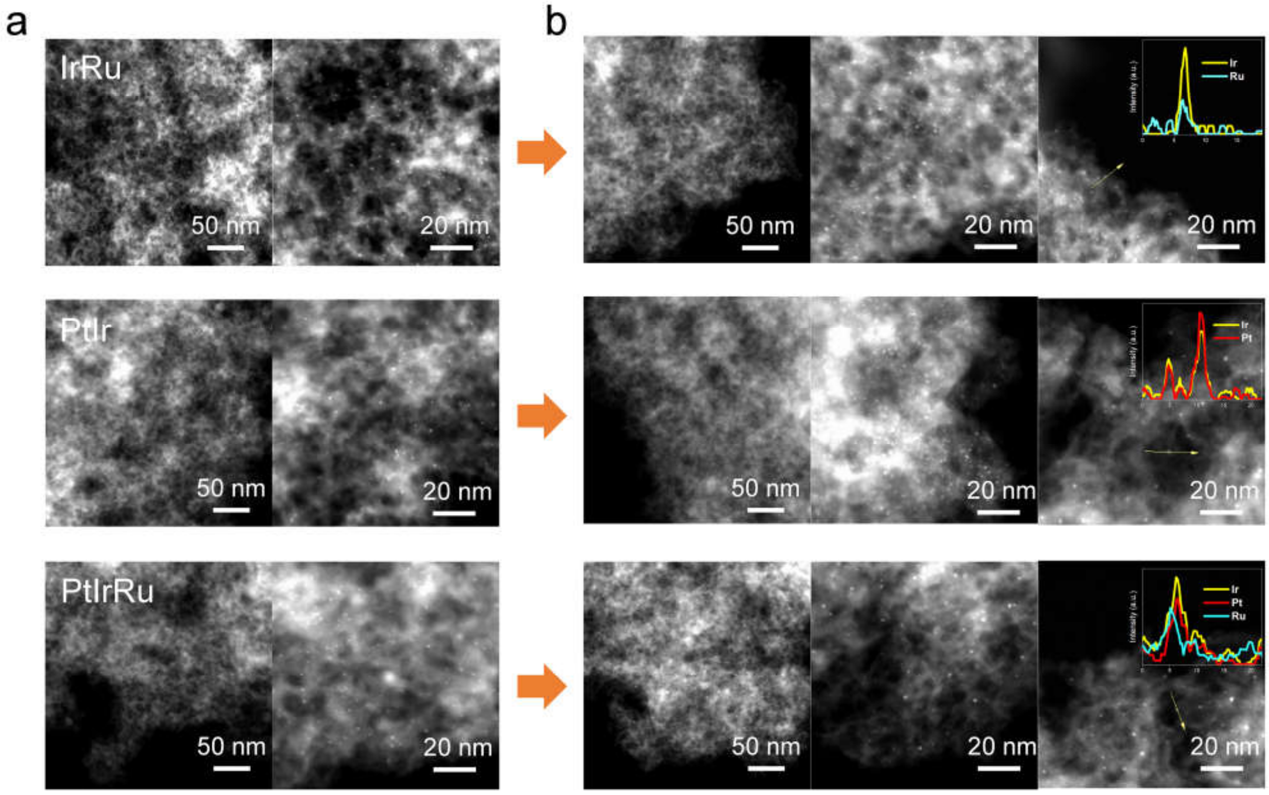

Supplementary Figure 15. HAADF-STEM images of bimetallic Pt-Ir and Ir-Ru and trimetallic Pt-Ir-Ru nanoclusters catalyst before (a) and after (b) sintering tests. Sintering test condition: 5% H_2/Ar, 700 °C, 600 min. Similar to the monometallic system, no any aggregation or overgrowth of bimetallic or tertiary nanoclusters were found, further demonstrating the generality of the sulfur-stabilizing method for synthesizing thermally stable multi-metallic nanoclusters.

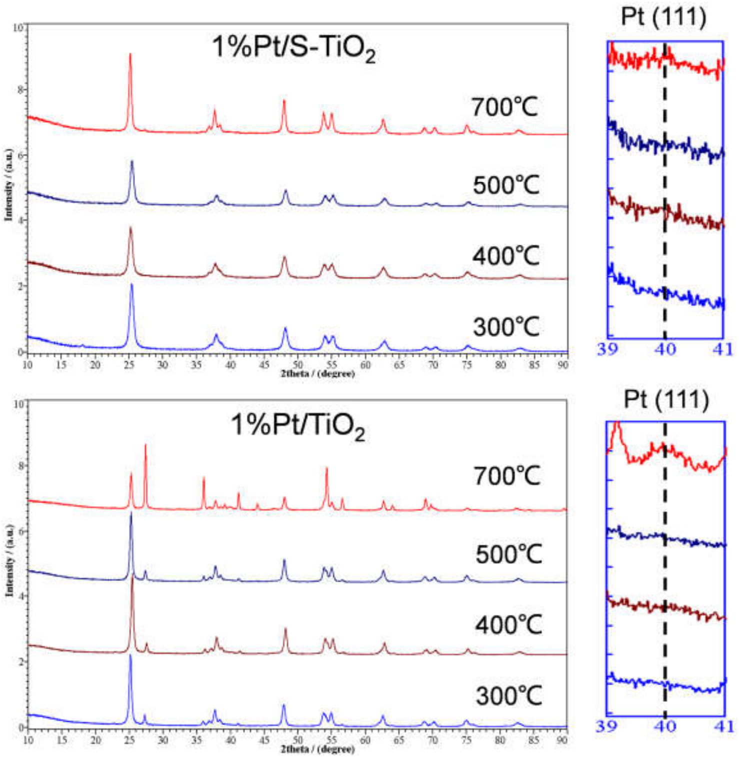

Supplementary Figure 16. XRD patterns of the Pt/S-TiO_2 and Pt/TiO_2 catalysts after annealing at different temperatures ranging from 300 to 700 °C) in 5% H_2/Ar for 120 min. The S-doped TiO_2 supports were prepared by pyrolysis of titanium tetraisopropanolate and thiourea[3]. Because the diffraction of TiO_2 supports is too strong, we did not observed any Pt diffraction peaks for the fresh catalysts with the low Pt loading of 1.0 wt%. After the sintering test at 700 °C, the Pt/TiO_2 catalyst gradually appeared Pt (111) diffraction peak, while the Pt/S-TiO_2 catalyst still remained unchanged.

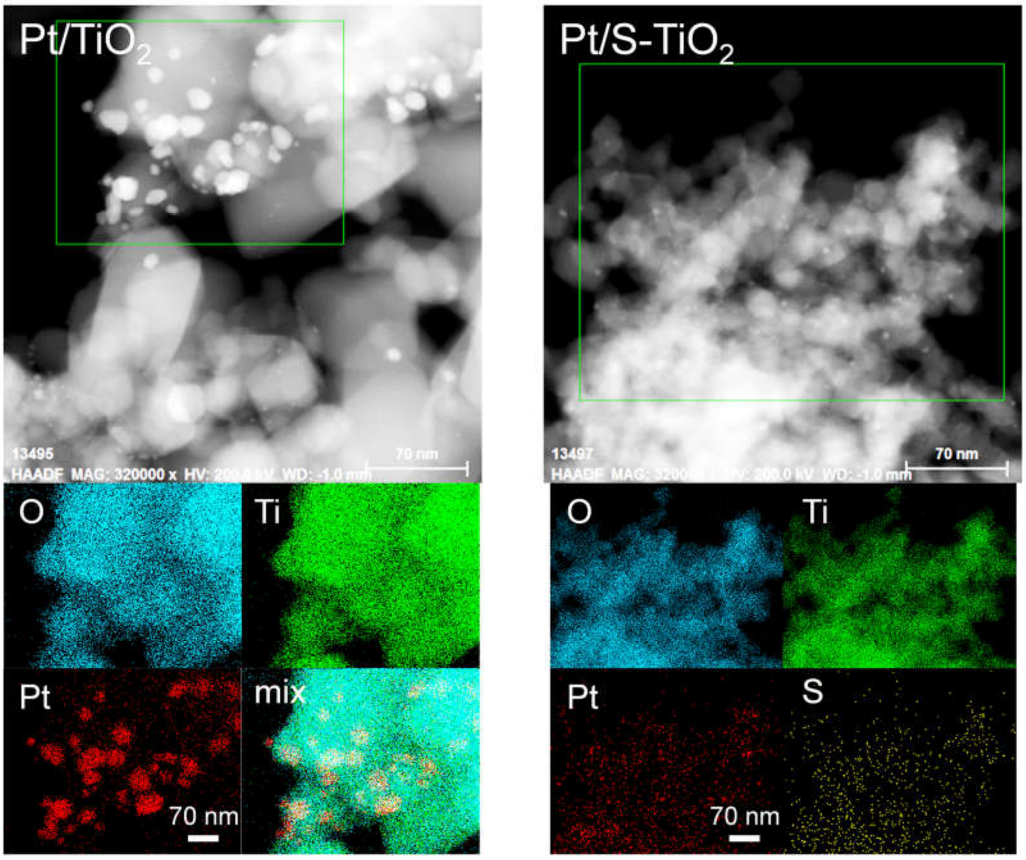

Supplementary Figure 17. HADDF-STEM images and EDS elemental mappings of the Pt/S-TiO_2 and Pt/TiO_2 catalysts after annealing at 700 °C in 5% H_2/Ar for 120 min, confirming the Pt sintering on Pt/TiO_2 and the anti-sintering on Pt/S-TiO_2.

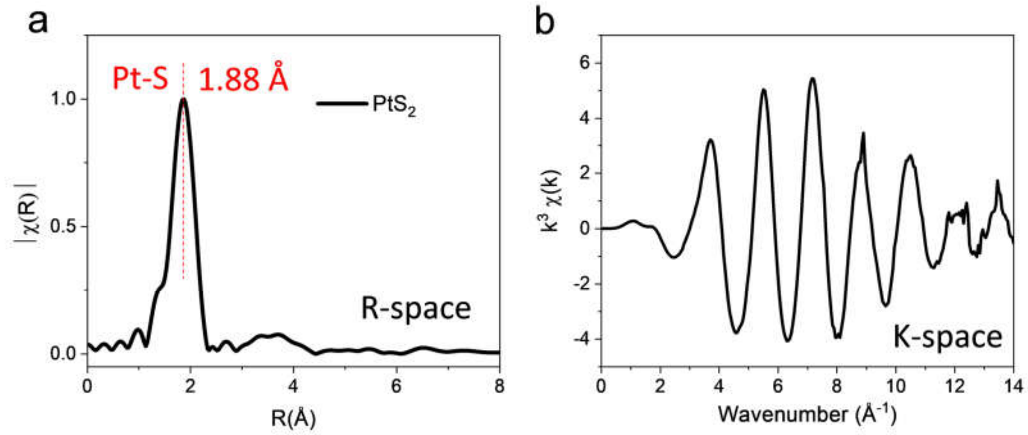

Supplementary Figure 18. R-space (a) and k-space (b) of k^3-weighted Pt L_3-edge of PtS_2. The k space of k^3-weighted Pt L_3-edge of PtS_2 shows only minor noise, suggesting the high data quality and guarantee the accuracy of Pt-S bond (about 1.88 Å).

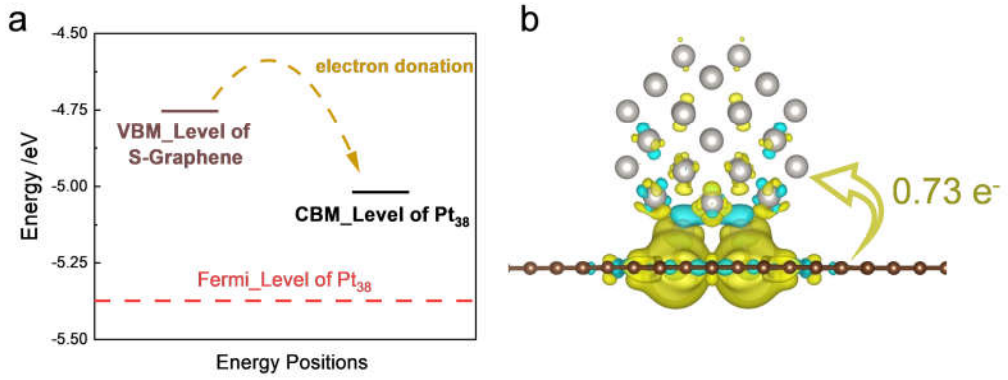

Supplementary Figure 19. (a) Illustration of the charge transfer direction based on the Frontier Orbital Theory. (b) Bader charge analysis results of S-Graphene supported Pt_{38} cluster.

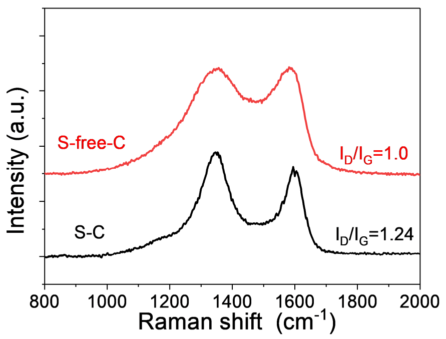

Supplementary Figure 20. Raman spectrum of S-C and S-free-C, indicating the higher graphitization of S-free-C after treatment at 1100 °C in 5% H_2/Ar for 120 min.

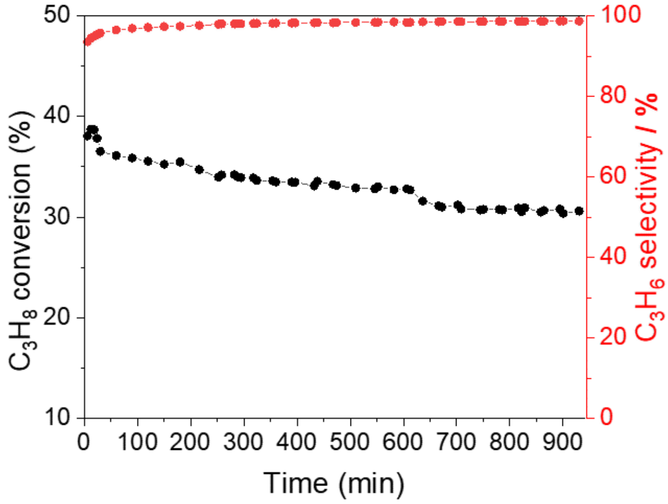

Supplementary Figure 21. Long-term stability test of PtSn/Al_2O_3 for PDH at 550 °C for 950 min. The selectivity of PtSn/Al_2O_3 (98%) was comparable to Pt/S-C, but the conversion on PtSn/Al_2O_3 catalyst gradually decreased from 39% to 32% (corresponding to 17.9% deactivation) after 600 min, then further slowly decayed to 30% after 950 min. The deactivation rate of PtSn/Al_2O_3 was 0.031 h^{-1} at same 600 min, which is also much higher than that of Pt/S-C (0.005 h^{-1}).

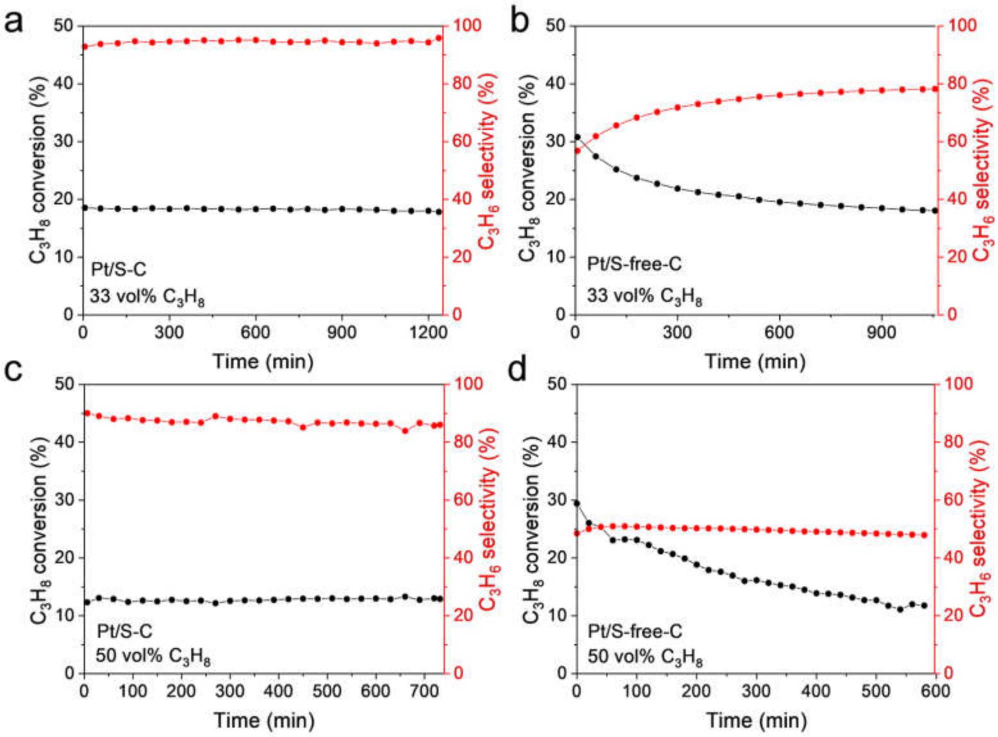

Supplementary Figure 22. (a-b) Catalytic performance of Pt/S-C (a) and Pt/S-free-C (b) under 33 vol% C_3H_8 (C_3H_8: H_2: Ar =1:1:1). (c-d) Catalytic performance of Pt/S-C (c) and Pt/S-free-C (d) under 50 vol% C_3H_8 (C_3H_8: H_2=1:1, no Ar dilution).

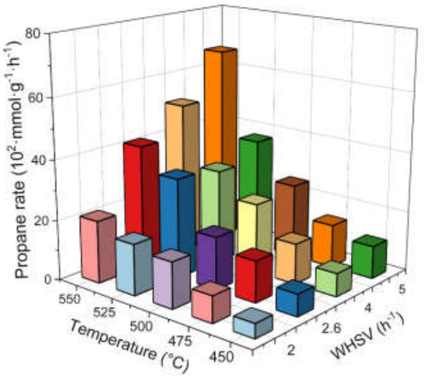

Supplementary Figure 23. Catalytic performances of Pt/S-C for PDH at different temperature and weight hourly space velocity (WHSV).

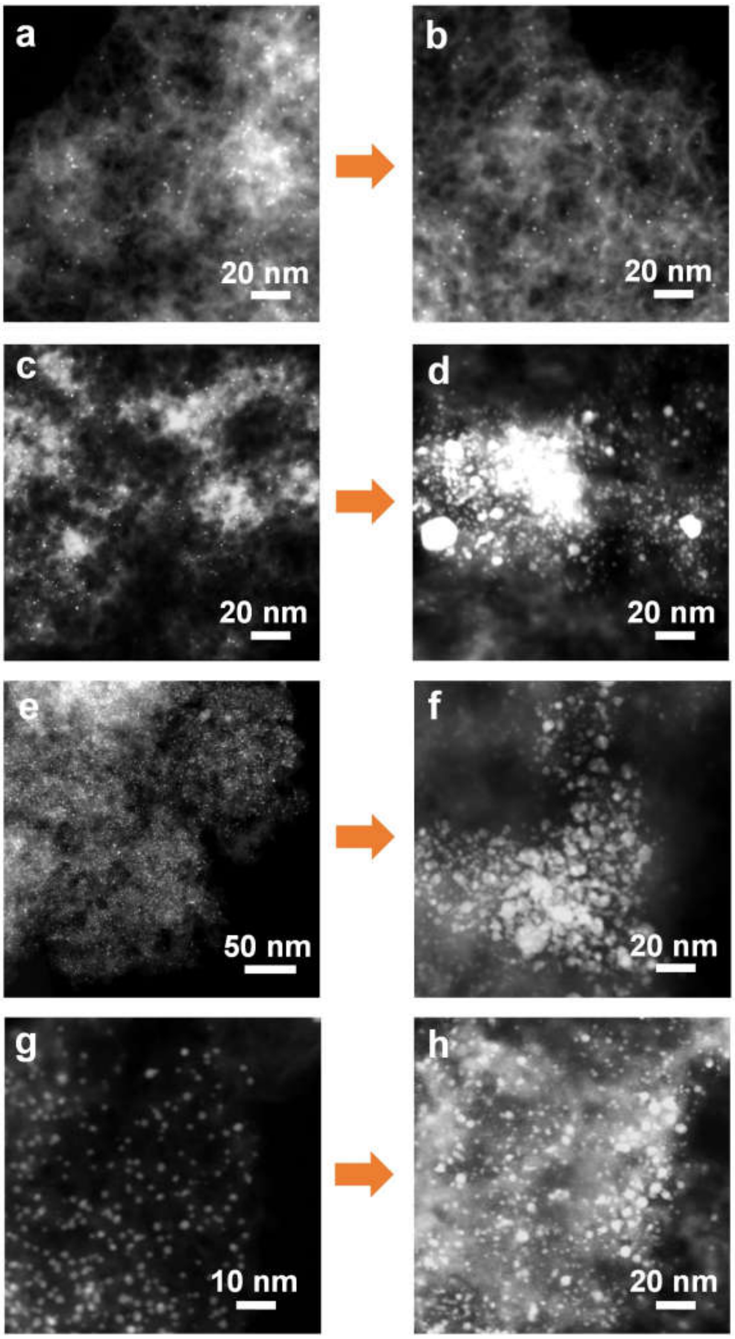

Supplementary Figure 24. HAADF-STEM images of fresh (a, c, e, g) and spent (b, d, f, h) of Pt/S-C (a, b), Pt/S-free-C (c, d), commercial Pt/C (e, f), and commercial Pt/Al_2O_3 (g, h), respectively. The spent catalysts were used in a 600 min PDH reaction.

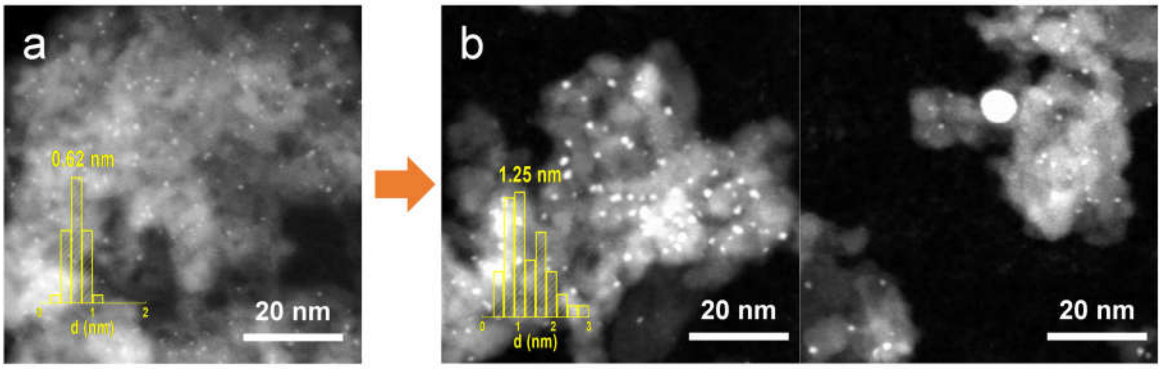

Supplementary Figure 25. HAADF-STEM images of fresh (a) and spent (b) of $PtSn/Al_2O_3$. The spent catalysts were used for PDH at 550 °C for 950 min.

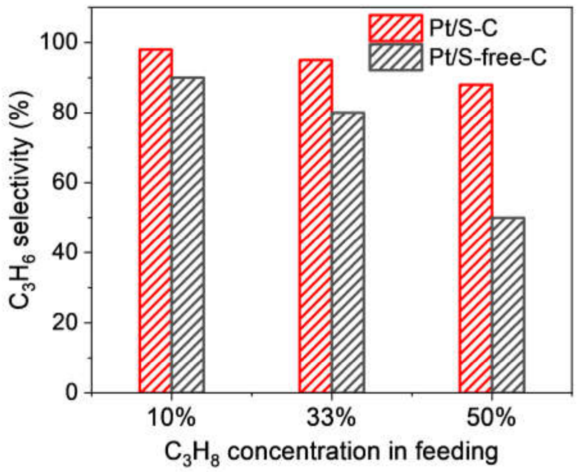

Supplementary Figure 26. The change of PDH selectivity under different propane concentration in reaction feeding.

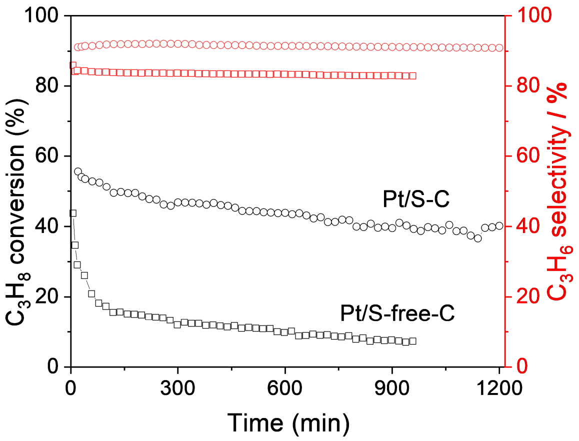

Supplementary Figure 27. The PDH reaction under higher 600 °C. Catalytic conditions: atmospheric pressure, 600 °C, C_3H_8/H_2= 1/1, with balance Ar for total flow rate of 15 mL min^{-1}, WHSV = 2 h^{-1} over 80 mg of sample.

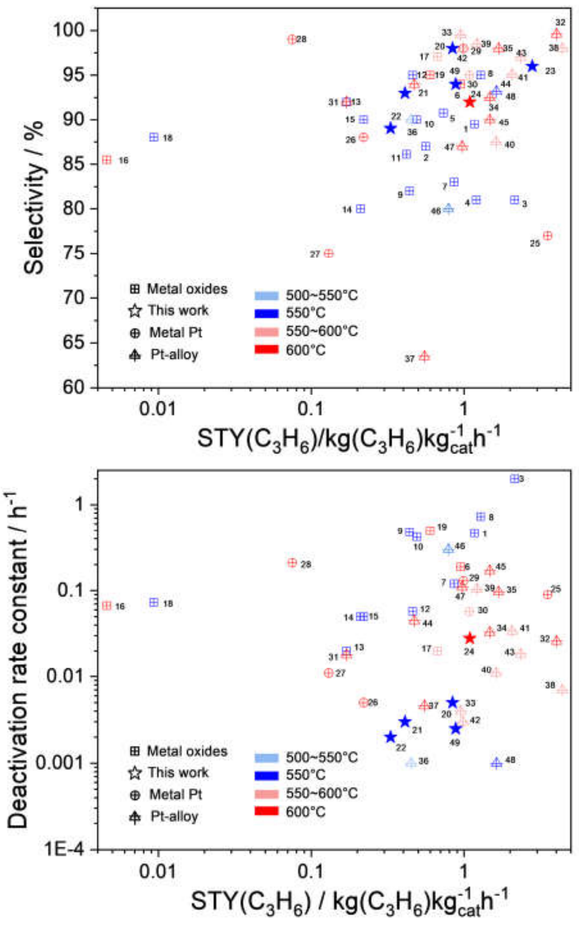

Supplementary Figure 28. Comparisons of the selectivity to propene, the space time yield of propene formation STY (C_3H_6) and deactivation rate over this work and other reported metal oxides, metal Pt and Pt-alloy catalysts for PDH. In order to ensure uniformity of catalytic conditions, the temperature is concentrated in 550°C~600°C, the propane content is under 10~50 vol % and the WHSV is around 1~5 h^{-1} except for a few samples. As can be seen, the Pt/S-C exhibited a lower STY (C_3H_6) values than partial metal oxides and most Pt-alloy at high C_3H_8 feed and only acquired a higher STY (C_3H_6) values under higher WHSV (5 h^{-1}) at 10 vol% C_3H_8 feed, which is superior to most monometallic Pt and partial Pt-alloys. As for the selectivity, there is little difference between Pt-alloy catalysts and the Pt/S-C, only some of metal oxides and monometallic Pt showed poorer selectivity. In terms of deactivation rate, the Pt/S-C is obviously in the top position with outstanding stability and even better than most Pt-alloys catalysts. Metal oxides catalysts show considerable activity, yet suffer from the loss of oxygen under reaction conditions, and rapid deactivation in a short time. Over the course of a catalytic cycle, due to the coke deposition, frequent regeneration operations are needed.

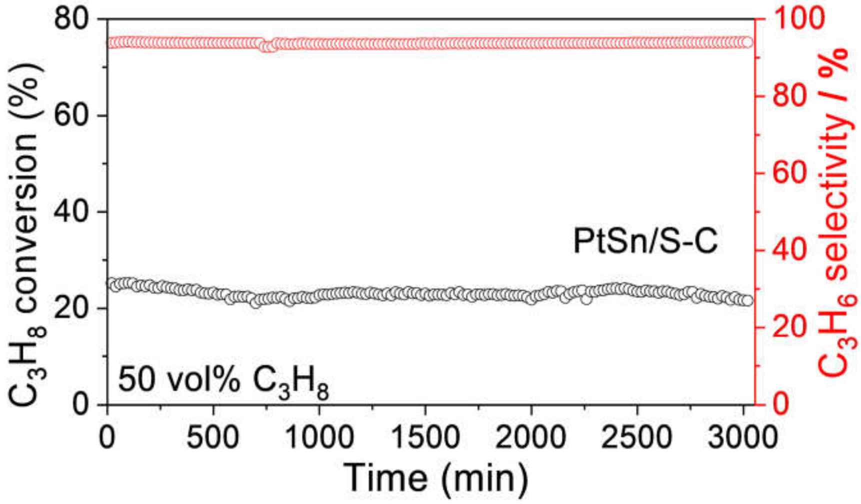

Supplementary Figure 29. Long-term stability test in PDH on Pt-Sn/S-C (Pt/Sn = 1). Catalytic conditions: 1 wt% Pt-Sn/S-C, atmospheric pressure, 550 °C, 50 vol% C_3H_8 feed, no Ar dilution, WHSV of propane = 3.9 h^{-1} over 60 mg of sample.

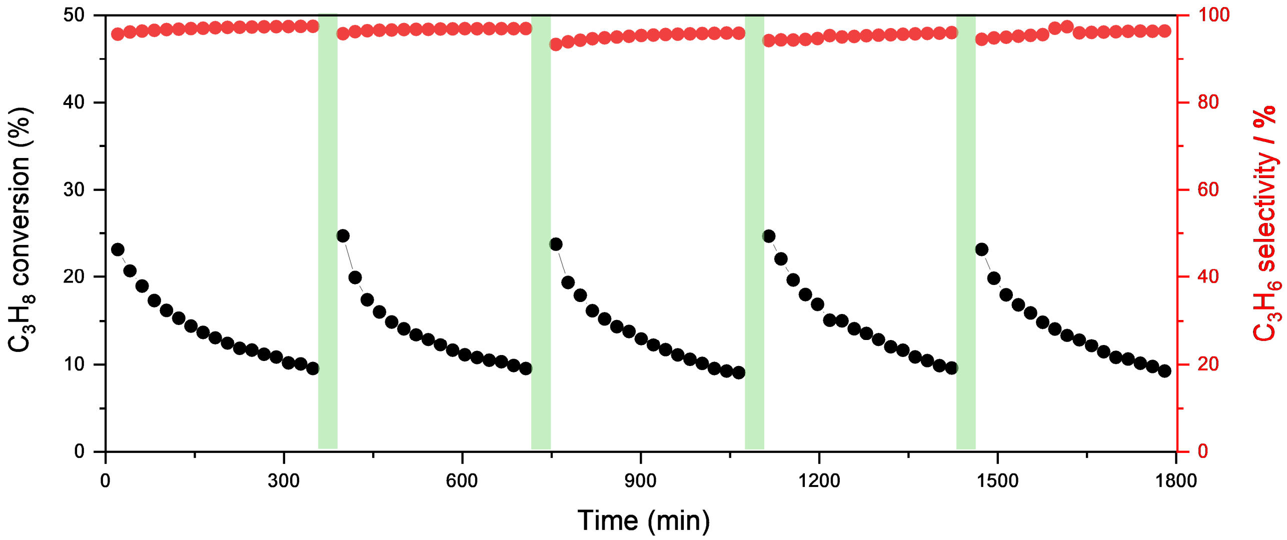

Supplementary Figure 30. Regeneration properties of Pt/S-TiO2 during the continuous cycles. The regeneration process was carried out under flowing air stream (25 mL min^{-1}) at 500 °C for 30 min. Catalytic conditions: 0.1 wt% Pt/S-TiO2, atmospheric pressure, 550 °C, C_3H_8/H_2= 1/1, with balance Ar for total flow rate of 20 mL min^{-1}, WHSV = 2 h^{-1} over 120 mg of sample.

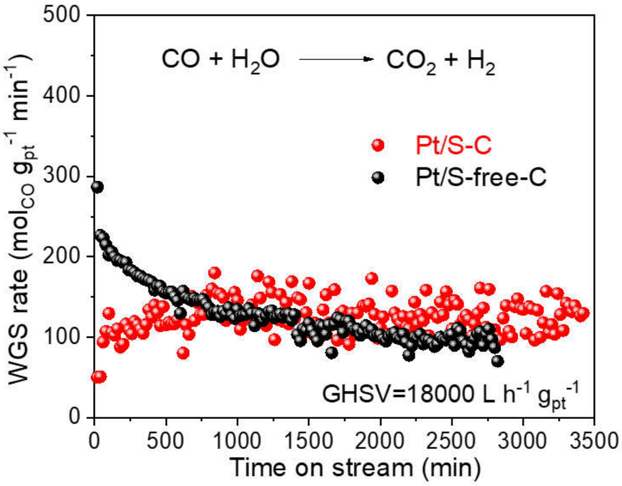

Supplementary Figure 31. Stability evaluation of the Pt/S-C and Pt/S-free-C catalysts in high-temperature WGS reactions. The catalytic performance of the catalysts in the WGS reaction was evaluated in a fixed-bed flow reactor. The catalyst (10 mg) was pretreated by 5% H_2/Ar at 200 °C for 2 h. After that, the temperature was increased to 400 °C, and the catalyst was exposed to the WGS reaction mixture. The reactant gas consisted of 5% CO (flow rate: 30 mL min^{-1}) and water vapor at 46 °C (water vapor pressure: 10.094 kPa) balanced with Ar that yielded the P_{CO}/P_{H2O} ratio of 1:2. All catalysts were heated to the desired reaction temperatures at a rate of 1 K min^{-1}, and the steady state compositions of the effluent gas were analyzed with an online gas chromatograph (FULI 9790II) with a TCD attached to a TDX column. The catalytic activity was calculated by the change in the CO concentrations of the inlet and outlet gases. The WGS rate was calculated based on the total Pt content. Although Pt/S-free-C exhibited a higher initial WGS activity, the rate rapidly decayed from a higher initial activity (286.7 $mol_{CO}g_{pt}^{-1}min^{-1}$) to 129.8 $mol_{CO}g_{pt}^{-1}min^{-1}$ after 600 min, which further decayed to 70.3 $mol_{CO}g_{pt}^{-1}min^{-1}$ after 2800 min. Encouragingly, the Pt/S-C catalyst exhibited a stable WGS rate about 129.8 $mol_{CO}g_{pt}^{-1}min^{-1}$ after continuous operation for 3500 min. The enhanced hydrothermal stability at high temperature further demonstrated the application potentials of the sulfur-stabilizing method for industrially relevant catalysis.

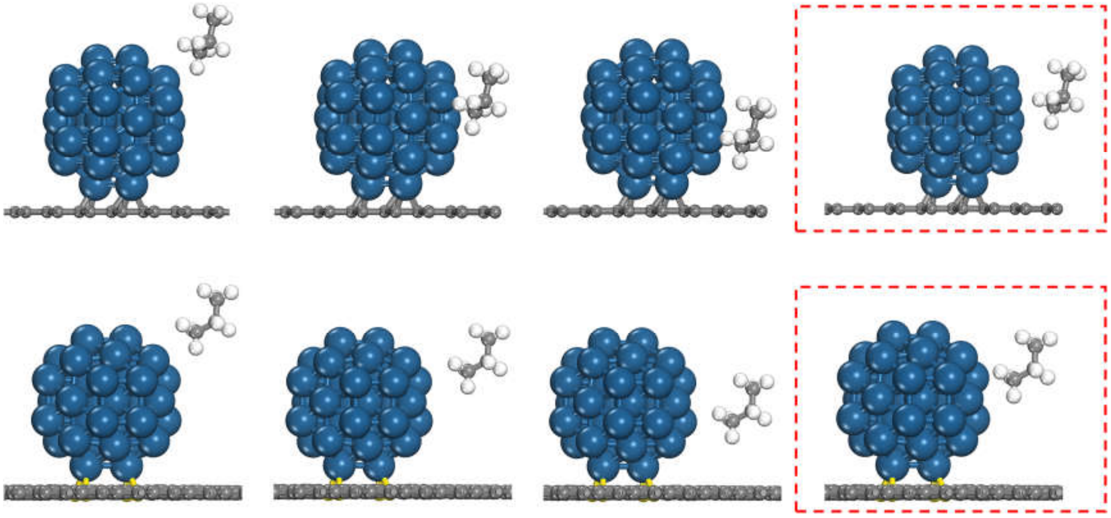

Supplementary Figure 32. The optimization of molecular adsorption sites. The red dotted frame is the spontaneously final stable state.

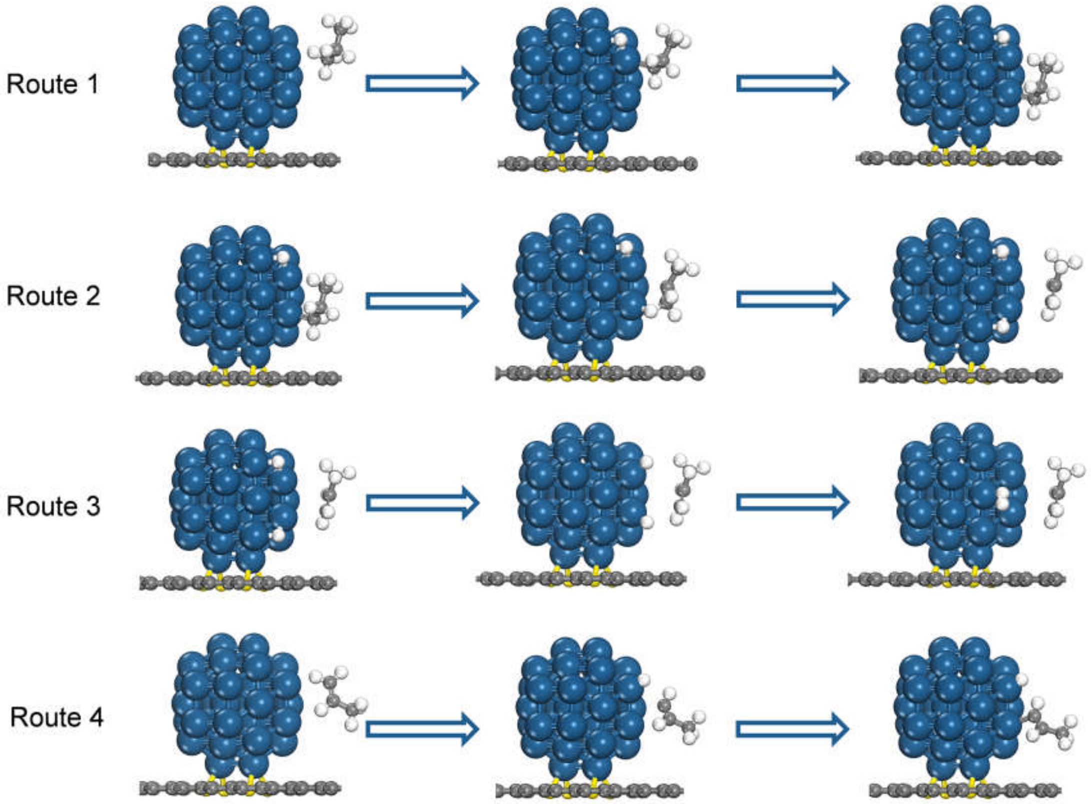

Supplementary Figure 33. PDH reaction paths on the Pt_{38}/S-Graphene.

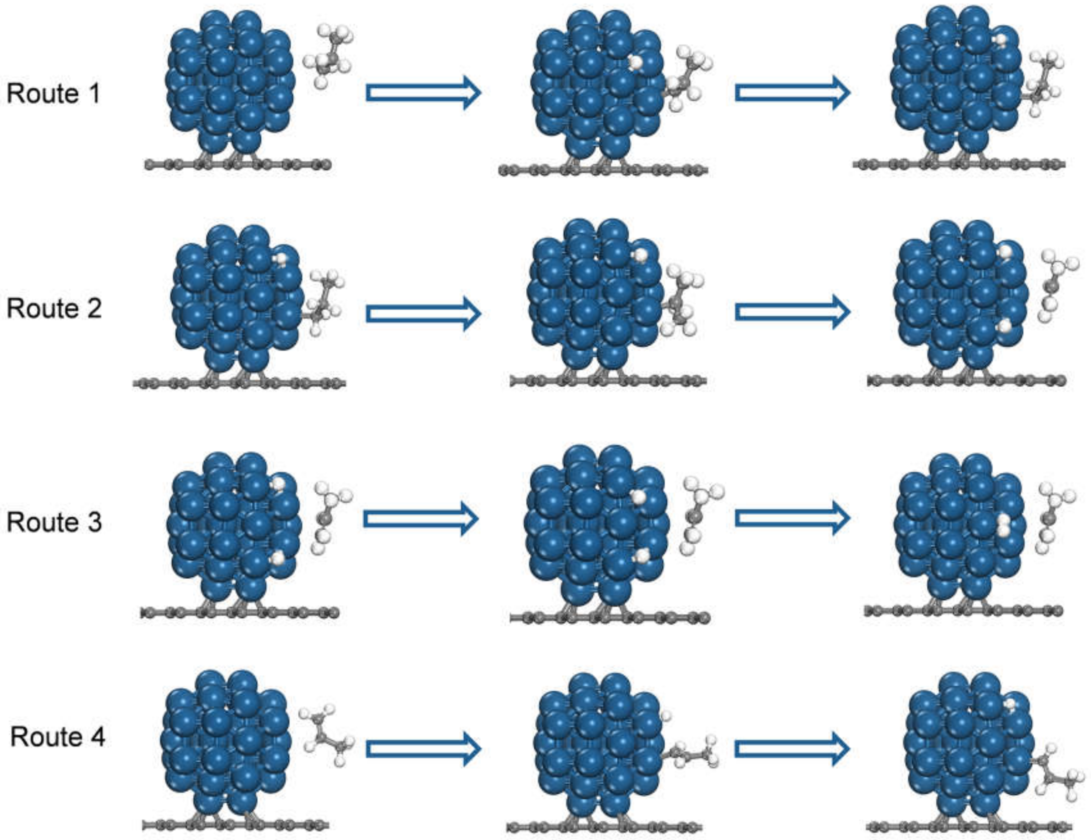

Supplementary Figure 34. PDH reaction paths on the Pt_{38}/Graphene.

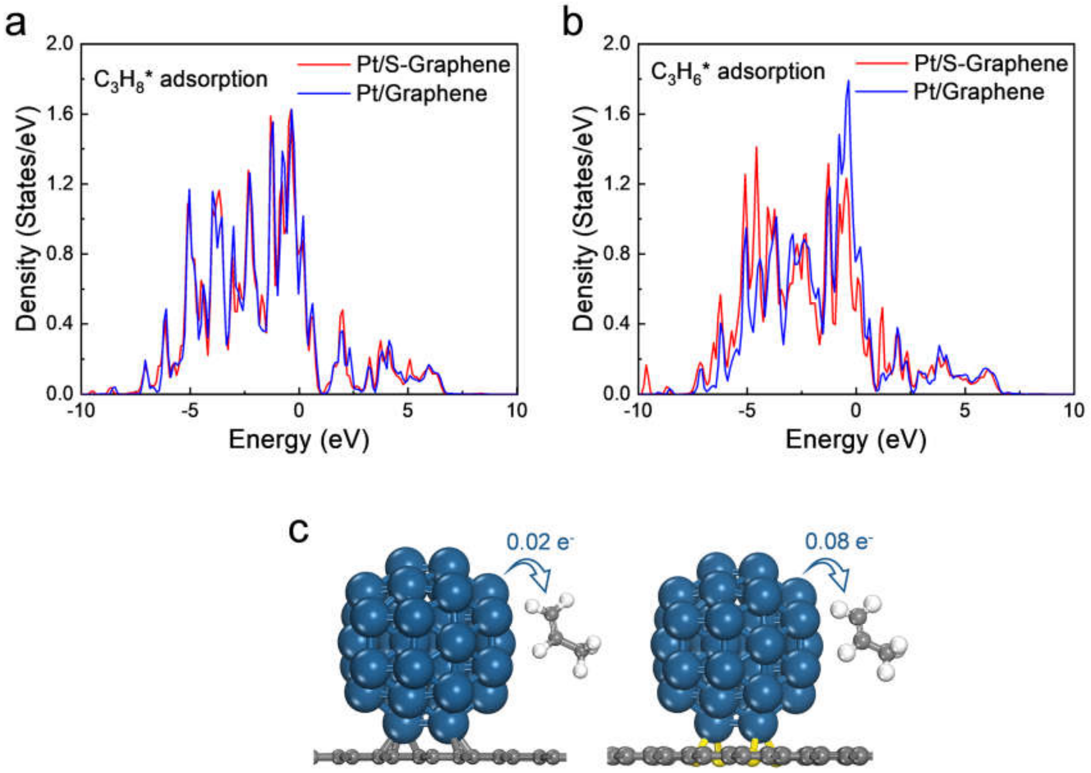

Supplementary Figure 35. Density of states (DOS) of (a) the C_3H_8* adsorption stage and (b) the C_3H_6* adsorption stage. (c) Bader charge analysis of the C_3H_6* intermediate adsorption stage on Pt_{38}/Graphene and Pt_{38}/S-Graphene.

Supplementary Table 1. Surface sulfur contents (determined by XPS) of different catalysts before and after thermal treatment at 700 °C for 600 min in 5% H_2/Ar.

Catalysts	Before treatment (at%)	After treatment (at%)
Ru/S-C	2.19	2.15
Rh/S-C	2.26	2.21
Os/S-C	2.31	2.28
Ir/S-C	2.22	2.24
Pt/S-C	2.49	2.45

Supplementary Table 2. Elemental analysis results of the S-C and S-free-C supports.

Supports	C (wt%)	N (wt%)	S (wt%)	O (wt%)
S-C	76.06	0.85	14.07	9.02
S-free-C	95.61	0.72	0.18	3.49

Supplementary Table 3. Summary of the catalytic performance of this work and other reported metal oxides, metal Pt and Pt-alloy catalysts for PDH.

No.		Catalysts	T (°C)	WHSV (h^{-1})	C_3H_8 Feed (vol. %)	STY(C_3H_6) $kg_{C3H6}kg_{cat}^{-1}$	Selectivity （%）	K_d (h^{-1})
1	Metal oxides	K–CrOx/Al_2O_3[4]	550	4.71	40	1.17	89.5	0.467
2		K–CrOx/Al_2O_3[5]	550	1.6	40	0.56	87	-
3		Cr_{10}ZrOx[4]	550	9.24	40	2.14	81	1.99
4		$Cr_{10}Zr_{90}/SiO_2$[6]	550	4.32	40	1.2	81	-
5		CrZrOx/SiO_2[7]	550	3.77	40	0.73	90.8	-
6		12VOx/Al_2O_3[8]	600	3.3	28	0.95	94	0.188
7		12V1Mg/Al_2O_3[9]	600	3.3	28	0.86	83	0.12
8		4Zn/TiZrOx[10]	550	4.71	40	1.28	95	0.73
9		0.05Ru/YZrOx[11]	550	1.57	40	0.44	82	0.48
10		Y_9Zr_{91}Ox[5]	550	1.6	40	0.49	90	0.422
11		m-ZrO_2[12]	550	1.89	40	0.42	86.1	-
12		2.5 wt% Cr-5 wt%Ni/Al[13]	550	1.08	10	0.46	95	0.058
13		4.3 wt%VOx/MCM-41[14]	550	1.78	40	0.17	92	0.02
14		4.3 wt%VOx/(10 wt% SiO_2 + 90 wt%Al_2O_3)[15]	550	1.78	40	0.21	80-94	0.05
15		Ga(i-Bu)$_3$/Al_2O_3[16]	550	1.35	20	0.22	90	0.05
16		5% In_2O_3–15%Ga_2O_3-80% Al_2O_3[17]	600	1.08	5	0.0046	85.5-56.8	0.067
17		Co–Al_2O_3–HAT[18]	590	2.9	20	0.67	97.1	0.02
18		Co/SiO_2[19]	550	6.38	20	0.0093	88-84	0.073
19		Zn/Al_2O_3[20]	600	3	28	0.6	95	0.5
20	This work	Pt/S-C	550	2.2	10	0.84	98	0.005
21			550	2.4	33	0.41	93	0.003
22			550	2.9	50	0.33	89	0.002
23			550	5	10	2.79	96	-
24			600	2.2	10	1.09	92	0.028
25		Pt/TA10[21]	600	10	26	3.52	77	0.09

26		0.28% Pt/ND@G[22]	600	1.6	5	0.22	88	0.005
27	Metal Pt	3.2 wt% Pt/CNT[23]	600	1.6	5	0.13	75	0.011
28		Pt/Mg(Ga)(Al)O[24]	600	2.6	20	0.075	99	0.21
29		Pt/Mg(In)(Al)O[25]	600	2.6	20	0.99	98	0.13
30		0.35 wt% Pt/Al_2O_3-nanosheet[26]	590	9.4	16	1.08	95	0.057
31		Pt_3In/SiO_2[27]	600	3	<50 (C_3H_8/H_2=1, balance N_2)	0.17	92	0.018
32		Pt_3Ga/CeAl[28]	600	10		4.02	99.6	0.026
33		0.42 wt% Pt0.42 wt% $Sn/MgAl_2O_4$[29]	580	2.2	10	0.95	99.5	0.004
34		PtSn/TS-1[30]	600	3	16	1.47	92.5	0.033
35		PtSnIn/08Zr-Al[31]	600	3.3	16	1.68	98	0.097
36		$0.1Pt10Cu/Al_2O_3$[32]	520	4.0	16	0.45	90	0.001
37		4.37 wt% Pt1.55 wt% Ga/SiO_2[33]	550	2	20	0.55	63.5	0.0046
38		$PtSn/Al_2O_3$ sheet[26]	590	9.4	16	4.39	98	0.007
39	Pt-based alloy	$PtSnAl_{0.2}$/SBA-15[34]	590	2.5	16	1.21	98.5	0.104
40		$Pt\text{-}Cu/MgAl_2O_4$[35]	590	6.8	47.5	1.62	87.5	0.011
41		$Pt\text{-}Ag/MgAl_2O_4$[35]	590	6.8	47.5	2.06	95.1	0.034
42		$Pt\text{-}Sn\text{-}5/MgAl_2O_4$[29]	580	2.4	10	0.98	98	0.003
43		0.5 wt% Pt0.9 wt% Sn/Al_2O_3 (A750)[36]	590	5.2	16	2.35	97	0.0185
44		$Pt–ZnO/Al_2O_3$[20]	600	3	28	0.472	94	0.045
45		$PtCu/Al_2O_3$[32]	600	4	16	1.47	90-94	0.17
46		$Pt\text{-}Sn/CeO_2$[37]	580	2.6	17	0.79	80-85	0.3
47		PtFe@Pt/SBA-15[38]	600	3.4	26	0.97	87	0.11
48		PtZn4@S-1[39]	550	3.6	25	1.63	93.2-99	0.001
49		Pt-Sn/S-C (this work)	550	3.9	50	0.88	94	0.003

Supplementary References

1 Liang, H. W. *et al.* Molecular metal-Nx centres in porous carbon for electrocatalytic hydrogen evolution. *Nat. Commun.* **6**, 7992 (2015).

2 Wu, Z.-Y. *et al.* Transition metal–assisted carbonization of small organic molecules toward functional carbon materials. *Sci. Adv.* **4**, eaat0788 (2018).

3 Ohno, T. *et al.* Preparation of S-doped TiO2 photocatalysts and their photocatalytic activities under visible light. *Appl. Catal. A-Gen.* **265**, 115-121 (2004).

4 Otroshchenko, T. P., Rodemerck, U., Linke, D. & Kondratenko, E. V. Synergy effect between Zr and Cr active sites in binary CrZrOx or supported CrOx/LaZrOx: Consequences for catalyst activity, selectivity and durability in non-oxidative propane dehydrogenation. *J. Catal.* **356**, 197-205 (2017).

5 Otroshchenko, T. P., Kondratenko, V. A., Rodemerck, U., Linke, D. & Kondratenko, E. V. Non-oxidative dehydrogenation of propane, n-butane, and isobutane over bulk ZrO 2-based catalysts: effect of dopant on the active site and pathways of product formation. *Catal. Sci. Technol.* **7**, 4499-4510 (2017).

6 Han, S. *et al.* Unraveling the Origins of the Synergy Effect between ZrO2 and CrO x in Supported CrZrO x for Propene Formation in Nonoxidative Propane Dehydrogenation. *ACS Catal.* **10**, 1575-1590 (2019).

7 Han, S. *et al.* The effect of ZrO2 crystallinity in CrZrOx/SiO2 on non-oxidative propane dehydrogenation. *Appl. Catal. A: Gen.* **590**, 117350 (2020).

8 Liu, G., Zhao, Z.-J., Wu, T., Zeng, L. & Gong, J. Nature of the Active Sites of VOx/Al2O3 Catalysts for Propane Dehydrogenation. *ACS Catal.* **6**, 5207-5214 (2016).

9 Wu, T. *et al.* Structure and catalytic consequence of Mg-modified VOx/Al2O3 catalysts for propane dehydrogenation. *AIChE J.* **63**, 4911-4919 (2017).

10 Han, S. *et al.* Elucidating the Nature of Active Sites and Fundamentals for their Creation in Zn-Containing ZrO2–Based Catalysts for Nonoxidative Propane Dehydrogenation. *ACS Catal.* **10**, 8933-8949 (2020).

11 Otroshchenko, T., Kondratenko, V. A., Rodemerck, U., Linke, D. & Kondratenko, E. V. ZrO2-based unconventional catalysts for non-oxidative propane dehydrogenation: Factors determining catalytic activity. *J. Catal.* **348**, 282-290 (2017).

12 Zhang, Y. *et al.* The effect of phase composition and crystallite size on activity and selectivity of ZrO2 in non-oxidative propane dehydrogenation. *J. Catal.* **371**, 313-324 (2019).

13 Li, P.-P. *et al.* The promotion effects of Ni on the properties of Cr/Al catalysts for propane dehydrogenation reaction. *Appl. Catal. A: Gen.* **522**, 172-179 (2016).

14 Sokolov, S., Stoyanova, M., Rodemerck, U., Linke, D. & Kondratenko, E. V. Comparative study of propane dehydrogenation over V-, Cr-, and Pt-based catalysts: Time on-stream behavior and origins of deactivation. *J. Catal.* **293**, 67-75 (2012).

15 Sokolov, S., Stoyanova, M., Rodemerck, U., Linke, D. & Kondratenko, E. Effect of support on selectivity and on-stream stability of surface VO x species in non-oxidative propane dehydrogenation. *Catal. Sci. Technol.* **4**, 1323-1332 (2014).

16 Szeto, K. C. *et al.* A strong support effect in selective propane dehydrogenation catalyzed by Ga (i-Bu) 3 grafted onto γ-alumina and silica. *ACS Catal.* **8**, 7566-7577 (2018).

17 Tan, S. *et al.* Propane dehydrogenation over In2O3–Ga2O3–Al2O3 mixed oxides. *ChemCatChem* **8**, 214-221 (2016).

18 Dai, Y. *et al.* γ-Al2O3 sheet-stabilized isolate Co2+ for catalytic propane dehydrogenation. *J. Catal.* **381**, 482-492 (2020).

19 Estes, D. P. *et al.* C–H Activation on Co, O Sites: Isolated Surface Sites versus Molecular Analogs. *J. Am. Chem. Soc.* **138**, 14987-14997 (2016).

20 Liu, G. *et al.* Platinum-Modified ZnO/Al2O3 for Propane Dehydrogenation: Minimized Platinum Usage and Improved Catalytic Stability. *ACS Catal.* **6**, 2158-2162 (2016).

21 Jiang, F. *et al.* Propane Dehydrogenation over Pt/TiO_2-Al_2O_3 Catalysts. *ACS Catal.* **5**, 438-447 (2015).

22 Liu, J. *et al.* Origin of the robust catalytic performance of nanodiamond–graphene-supported Pt nanoparticles used in the propane dehydrogenation reaction. *ACS Catal.* **7**, 3349-3355 (2017).

23 Liu, J. *et al.* Defect-driven unique stability of Pt/carbon nanotubes for propane dehydrogenation. *Appl. Surf. Sci.* **464**, 146-152 (2019).

24 Siddiqi, G., Sun, P., Galvita, V. & Bell, A. T. Catalyst performance of novel Pt/Mg (Ga)(Al) O catalysts for alkane dehydrogenation. *J. Catal.* **274**, 200-206 (2010).

25 Xia, K., Lang, W.-Z., Li, P.-P., Yan, X. & Guo, Y.-J. The properties and catalytic performance of PtIn/Mg (Al) O catalysts for the propane dehydrogenation reaction: Effects of pH value in preparing Mg (Al) O supports by the co-precipitation method. *J. Catal.* **338**, 104-114 (2016).
26 Shi, L. *et al.* Al_2O_3 Nanosheets Rich in Pentacoordinate Al^{3+} Ions Stabilize Pt-Sn Clusters for Propane Dehydrogenation. *Angew. Chem. In. Ed.* **54**, 13994-13998 (2015).
27 Zha, S. *et al.* Identification of Pt-based catalysts for propane dehydrogenation via a probability analysis. *Chem. Sci.* **9**, 3925-3931 (2018).
28 Wang, T. *et al.* Effects of Ga doping on Pt/CeO2-Al2O3 catalysts for propane dehydrogenation. *AIChE J.* **62**, 4365-4376 (2016).
29 Zhu, H. *et al.* Sn surface-enriched Pt–Sn bimetallic nanoparticles as a selective and stable catalyst for propane dehydrogenation. *J. Catal.* **320**, 52-62 (2014).
30 Li, J. *et al.* Size effect of TS-1 supports on the catalytic performance of PtSn/TS-1 catalysts for propane dehydrogenation. *J. Catal.* **352**, 361-370 (2017).
31 Long, L.-L. *et al.* The comparison and optimization of zirconia, alumina, and zirconia-alumina supported PtSnIn trimetallic catalysts for propane dehydrogenation reaction. *J. Ind. Eng. Chem.* **51**, 271-280 (2017).
32 Sun, G. *et al.* Breaking the scaling relationship via thermally stable Pt/Cu single atom alloys for catalytic dehydrogenation. *Nat. Commun.* **9**, 4454, doi:10.1038/s41467-018-06967-8 (2018).
33 Searles, K. *et al.* Highly productive propane dehydrogenation catalyst using silica-supported Ga–Pt nanoparticles generated from single-sites. *J. Am. Chem. Soc.* **140**, 11674-11679 (2018).
34 Fan, X. *et al.* Dehydrogenation of propane over PtSnAl/SBA-15 catalysts: Al addition effect and coke formation analysis. *Catal. Sci. Technol.* **5**, 339-350 (2015).
35 Ren, G.-Q. *et al.* Effect of group IB metals on the dehydrogenation of propane to propylene over anti-sintering Pt/MgAl2O4. *J. Catal.* **366**, 115-126 (2018).
36 Jang, E. J., Lee, J., Jeong, H. Y. & Kwak, J. H. Controlling the acid-base properties of alumina for stable PtSn-based propane dehydrogenation catalysts. *Appl. Catal. A: Gen.* **572**, 1-8 (2019).
37 Xiong, H. *et al.* Thermally stable and regenerable platinum–tin clusters for propane dehydrogenation prepared by atom trapping on ceria. *Angew. Chem. In. Ed.* **129**, 9114-9119 (2017).
38 Cai, W. *et al.* Subsurface catalysis-mediated selectivity of dehydrogenation reaction. *Sci. Adv.* **4**, eaar5418 (2018).
39 Sun, Q. *et al.* Subnanometer Bimetallic Platinum-Zinc Clusters in Zeolites for Propane Dehydrogenation. *Angew. Chem. In. Ed.* **59**, 19450-19459, doi:10.1002/anie.202003349 (2020).
